# Evaluating paratransgenesis using engineered symbiotic bacteria for *Plasmodium* inhibition in mosquito vectors: A systematic review

**DOI:** 10.1371/journal.pntd.0013654

**Published:** 2026-02-12

**Authors:** Wisdom Deborah Cleanclay, Fabrice Banadzem Kernyuy, Irrinus Fonyuy Kintung, Nina Ghislaine Yensii, Joan Amban Chick, Agnes Mbiaya Mbeng Obi

**Affiliations:** 1 Department of Biochemistry, Covenant University, Ota, Ogun State, Nigeria; 2 Covenant Applied Informatics and Communication Africa Centre of Excellence (CApIC-ACE), Covenant University, Ota, Ogun State, Nigeria; 3 Covenant University Public Health and Wellbeing Research Cluster (CUPHWERC), Covenant University, Ota, Ogun State, Nigeria; 4 Department of Computer and Information Sciences, Covenant University, Ota, Ogun State, Nigeria; 5 Department of Microbiology, Covenant University, Ota, Ogun State, Nigeria; Yale University School of Public Health, UNITED STATES OF AMERICA

## Abstract

Malaria is a significant health problem in the world and has been increased by the emerging resistance to insecticides and antimalarial drugs. New measures must therefore be implemented as an emergency to break the cycle of *Plasmodium* parasite transmission by the *Anopheles* mosquitoes. This systematic review assessed the effectiveness of paratransgenesis, an engineering approach that utilizes symbiotic microbes to deliver antiplasmodial molecules into the midgut of the mosquito as a transmission-blocking agent. PubMed, ScienceDirect, and Web of Science were searched in accordance with the PRISMA guidelines, yielding 1,289 records. Ten eligible studies were then included after screening. The chosen articles studied bacterial and fungal symbionts, such as *Asaia, Serratia, Pantoea, Enterobacter,* and *Aspergillus oryzae,* that have been engineered to produce effector proteins, such as Scorpine, EPIP, Defensin, and SM1–2 peptides. The delivery of oral sugar meals was always associated with colonization of the mosquito midguts, and results reported high levels of inhibition of oocysts or sporozoites in the mosquitoes. Scorpine was the strongest and most commonly used effector with a high level of up to 97.8% inhibition of *P. falciparum* oocysts in various microbial systems. The combination of two or multiple-effector approaches increased the efficacy in some cases, surpassing 89% parasite inhibition. The risk of bias measurement showed moderate variation in the methods, yet it was in favor of the sound findings. All evidence suggests that paratransgenesis is a potentially important malaria control tool, complementing existing approaches to malaria control. Nevertheless, ecological safety, microbial stability, and field validation are the key obstacles before the translation to large-scale use.

## Author summary

Malaria is one of the key world health problems, especially in sub-Saharan Africa, where the rising resistance to insecticides and antimalarial drugs is posing a threat to the current control mechanisms. New strategies are urgently needed to break the cycle of the spread of Plasmodium parasites by the Anopheles mosquito. A potential solution is paratransgenesis, which involves the genetic modification of naturally occurring mosquito-associated microbes to produce molecules that prevent the development of parasites in the gut of the mosquito. In this systematic review, we examined experimental studies that investigated paratransgenesis as a malaria control measure. Using the PRISMA guideline, we identified ten eligible studies that had engineered bacterial or fungal symbionts to express antiplasmodial effector proteins. These studies showed that feeding mosquitoes a sugar meal containing engineered microbes resulted in a stable colonization of the mosquito midgut. Several of the molecules tested were highly effective in reducing parasite development, with scorpine consistently showing strong transmission-blocking activity, achieving parasite inhibition rates greater than 90% in different experimental systems. Inhibitory effects were also further augmented by the combination of several effector molecules. Overall, the finding suggests that paratransgenesis has strong potential as an additional malaria control measure. Nevertheless, contests concerning ecological safety, microbial stability, and field implementation will need to be solved before large-scale implementation can be done.

## Introduction

Malaria is an illness that is life-threatening with significant implications to the human genome, as exemplified by the selection of genetic adaptive mechanisms, including sickle cell trait and beta-thalassemia [1]. *Plasmodium* parasites cause malaria, a potentially deadly infectious disease, and are transmitted to humans as a result of being bitten by infected female *Anopheles* mosquitoes [2,3]. *P. malariae*, *P. vivax*, *P. ovale*, and *P. falciparum* are *Plasmodium* species that cause human infection depending on their severity in different geographical areas [4]. Of them, *P. falciparum* is the deadliest as it multiplies extremely fast in the bloodstream and mainly affects African children, causing severe anemia and the highest rates of deaths related to malaria [5,6]. The disease remains one of the greatest issues of public health concern, particularly in low-income countries, and poses serious mortality and morbidity, particularly in sub-Saharan Africa [7–9]. In 2023, the number of malaria cases was 263 million cases in 83 countries with malaria, and this figure is one million higher than in the previous year [10]. The transition was mostly witnessed in the African and Eastern Mediterranean regions, with the highest increase in cases in Nigeria, the Democratic Republic of Congo, Niger, Tanzania, Ethiopia, and Madagascar [10]. Although there are many tools to prevent and control malaria, such as insecticide-treated bed nets, indoor residual spraying, chemoprevention, and the malaria vaccine, the malaria prevention process is getting more difficult because there are insecticide-resistant mosquitoes and *Plasmodium* strains that are resistant to drugs [11–13]. The low effectiveness of the existing interventions, as well as the lack of a long-term vaccine, emphasize the inadequacy of new approaches in malaria prevention [14]. Recent studies have presented the mosquito gut microbiome as a potential target to control malaria at the parasitic level, which is arguably a more successful approach than traditional antimalarial drugs and insecticides [15,16].

The microbes obtained from the gut of mosquitoes have been shown to play a significant role in regulating the development of the *Plasmodium* parasite through an immune response that is also controlled. Malaria transmission depends on the bacterial species that favor or discourage the survival of parasites [17]. The notable ones are *Wolbachia spp., Serratia spp.,* and *Asaia spp*. due to their ability to produce bioactive compounds that inhibit the development of *Plasmodium*. Some also strengthen the natural immunity of the mosquito, creating an unfavorable environment for the parasite [18].

The activation of immune signaling pathways in the mosquito is a primary mechanism by which the gut microbiota affects the development of *Plasmodium*. These include Immune Deficiency (IMD), Toll, and the Janus Kinase-Signal Transducer and Activator of Transcription (JAK-STAT) pathways. The stimulation of these pathways leads to the synthesis of antimicrobial peptides (AMPs) that can identify and destroy attacking pathogens [18,19]. These pathways are directly induced by some gut bacteria, which indirectly inhibit the survival of *Plasmodium* [17,20]. The mosquito midgut is another important defense mechanism that is dependent on competition for resources. The rapid bacterial growth that follows a blood meal causes activation of a systemic immune response. This immune response and microbial growth decrease the supply of vital nutrients needed in the growth of *Plasmodium* [21]. Along with competition among nutrients, the secretion of gut bacteria into inhibitory compounds also disrupts the development and transmission of parasites [21,22]. In addition, the connections among the microbiota of the mosquito and other pathogens also indicate a complex regulation mechanism. An example of such is the alteration of the composition and functioning of the gut microbiota by viral infections, which implies that various pathogens can affect the existence of the other and the immune landscape of the host [23]. Even with an increased understanding, a number of regulatory processes are not well comprehended. One such case is that Peptidoglycan Recognition Proteins (PGRPs) are known to induce the IMD pathway, but its direct role in the regulation of Thioester-containing protein 1 (TEP1), an important effector in the clearance of *Plasmodium,* is not well understood [17,24,25]. This uncertainty shows that there are more unknown immune regulatory networks that should be explored.

Paratransgenesis is a vector control method where genetic engineering is used to incorporate effector molecules in a symbiotic microorganism, like a bacterium, virus, or fungus normally associated with insects, like a mosquito, to express the effector molecules in the mosquito midgut, which disrupts the development and spread of the parasites [26,27]. The symbiote microbes are engineered to produce effector molecules, and they are reintroduced into the mosquito where they cause the desired effect, e.g., interrupting the life cycle of the disease organism [28,29]. Knowledge of the mosquito microbiota is also important in determining the success of a paratransgenic approach, especially in the identification of bacterial strains that are strictly attached to mosquitoes and can be transferred to their offspring [30]. The high capacities and ranges of engineered microbes to infect and disseminate among a diverse variety of mosquito species are also a strong suit of using them as vectors to control disease vectors, as compared to the genetically engineered mosquitoes, which have to be adapted to each specific species of the target vectors to effectively block the pathogen. There is more applicability and scalability with engineered bacteria, fungi, or viruses. The manufacturing of these microbes on a large scale is typically easier and less expensive than the complicated and resource-intensive process of rearing transgenic lines of mosquitoes to be used out of the field [27].

Moreover, engineered microbes can quickly replicate in mosquito hosts and be transmitted horizontally (mosquito to mosquito) and vertically (from parent to offspring), effectively spreading across the populations of vectors and increasing their potential to control disease on a large scale [31]. On the other hand, it is quite difficult to manipulate the genomes of the eukaryotic organisms, including mosquitoes. These include the possibility of unwanted genetic mutations, changes in allele frequencies, and the possibility of developing pathogen resistance [25]. This has led to increased consideration of the disease control strategies based on the utilization of microbial symbionts as a more feasible and scalable alternative.

Paratransgenesis depends on the biological compatibility, genetic stability, and ecological viability. First, the microorganism, which is usually a culturable, symbiotic, or commensal bacterium, should be a natural part of the mosquito and possess genetic engineering capabilities [32]. To achieve sustained effects throughout the life cycle of the mosquito, the bacterium must be capable of surviving in all life cycle stages, such as the metamorphosis between the larval stage and adult stage, a characteristic referred to as transstadial transmission. This capability is manifested by bacteria such as *Asaia* [33]. It should also be safe to humans and animals, and must be capable of colonizing as many species of mosquitoes as possible, and its ability to maintain its natural fitness and stability after genetic modification [29].

One of the essential elements of such a system is the process of recognition of an effector molecule, a substance that interferes with the growth of pathogens. Such a molecule will either need to be expressed or delivered to the surface of the bacterium and manufactured in adequate quantities [29,34]. This means that not only is it necessary for the bacterium to survive in the mosquito host over a long period of time, but also that it is able to remain metabolically active in order to produce the intended inhibitory effect. An important benefit of microbial-based vector control over genetically modified mosquitoes is the extended host range and infections with engineered microbes. Transgenic insects can be used to infect and transmit pathogens across various species of mosquitoes, but transgenic mosquitoes need to be adapted to each species of vector to efficiently prevent the spread of pathogens. Genetic modification of microbes in bulk is less complicated and less expensive than raising huge transgenic mosquitoes to release them in the field [27].

Genetically modified bacteria have been shown to interfere with or block *Plasmodium* development in mosquitoes through the use of proof-of-concept studies [35–37]. The genera *Asaia, Pantoea, Serratia, Pseudomonas,* and *Escherichia coli* are some of the symbiotic bacteria found in malaria vectors and have been widely explored as possible vectors for malaria-controlling interventions in paratransgenesis. The main objective of this systematic review is to assess the usefulness of paratransgenesis as a method of malaria control with emphasis on the use of the method to disrupt the life cycle of the *Plasmodium* parasite in the mosquito host. Precisely, the review will attempt to provide the responses to the following research question: In *Anopheles* mosquitoes infected with *Plasmodium spp.*, how effective is paratransgenesis with engineered symbiotic microorganisms compared to non-modified symbionts in reducing parasite development and transmission?

## Materials and methods

### Protocol and registration

The protocol was registered in PROSPERO, with the identification number CRD420251130464 [38].

### Reporting standards

This systematic review was conducted in accordance with the Preferred Reporting Items for Systematic Reviews and Meta-Analysis (PRISMA) guideline [39].

### Study period and location

The literature search, data screening, and analysis were conducted in May and June 2025 at the Department of Biochemistry, College of Science and Technology, Covenant University, Nigeria.

### Search strategy

In the selection of the studies, we adhered to the PRISMA guidelines for a systematic and transparent process. The literature review was undertaken in the three databases: PubMed (https://pubmed.ncbi.nlm.nih.gov/), ScienceDirect (https://www.sciencedirect.com/), and Web of Science (https://www.webofscience.com), to identify studies on the role of the mosquito gut microbiome in the development of *Plasmodium* parasites and the transmission of malaria. The search strategy was an integration of the relevant keywords and Boolean operators, which are: (“Mosquito microbiome” OR “Mosquito gut microbiota”) AND (“Plasmodium parasite development” OR “Plasmodium parasite inhibition”) AND (“Microbiome engineering” OR “Microbiome manipulation”) AND (“Plasmodium transmission” OR “Malaria transmission”) AND (“Paratransgenesis” OR “Genetically modified mosquitoes” OR “Antiplasmodial metabolites”). The search in PubMed and ScienceDirect was carried out under the “All Fields” filter to guarantee that the most relevant papers were obtained. Web of Science database, on the contrary, was carried out under the “Topic” field, which included the title, abstract, and keywords to reduce the search results to more relevant publications. The date filter in all the databases was from “2000/01/01” to “2025/05/01”.

### Inclusion and exclusion criteria

The inclusion criteria for this review were properly structured to include studies that directly addressed the role of the mosquito gut microbiome in impacting *Plasmodium* parasite development and malaria transmission. The articles that were considered to be eligible were original research articles published between 2000 and 2025, written in English Language because most of the reviewers and readers have English language proficiency and most of the relevant research in this area is published in English. Research was required to concentrate on the interplay between the mosquito microbiota and *Plasmodium spp*., or manipulation of the microbiome community such as paratrangenesis, or the use of genetically engineered mosquitoes to prevent malaria infection. Research studies were also required to give accounts on experimental procedures and quantifiable results in regard to parasite inhibition or transmission-blocking activity. The exclusion criteria comprised review articles, editorials, abstracts of conferences, studies not focused on the mosquito microbiomes, articles with no experimental data or methodological description, and any articles that did not cover any of the aspects of *Plasmodium* development and transmission. The extensive filtering would ensure that only high-quality studies of specific relevance were incorporated in the final synthesis.

### Study selection process and data extraction

The process of selecting studies was done in an extensive and organized manner. Three databases were used, with search terms mentioned above, and an initial 1,289 records were retrieved. The retrieved articles were uploaded into Rayyan AI in order to automatically identify duplicates. These duplicates were manually screened and resolved, then titles and abstracts were screened, and lastly, a full-text screening. Three reviewers were then used to confirm and thoroughly scrutinize the articles. The articles that were collected were independently selected by two researchers based on the methods, results, and analyses that were employed. The decisions were also reviewed in a cross-check by another reviewer to ascertain accuracy and consistency, and any deviations in decisions were clarified by discussion. As a result of this procedure, 10 original research articles were chosen and included in the qualitative synthesis. The selection process is represented in the PRISMA flow diagram. A data extraction form was developed to identify the essential features of a study such as author(s), year of publication, objective/aim, mosquito species, *Plasmodium* species, plasmid used, bacteria/fungi paratransgenized, expression of the effector protein, bacterial dose, bacteria delivery mode, dissection time, colonization verified, and result(s) of *Plasmodium* inhibition.

### Risk of bias analysis

In order to ensure the internal validity of the synthesized findings, a structured risk of bias (RoB) evaluation was performed with the help of a modified version of the SYRCLE RoB tool, which was specifically created to consider animal intervention studies. This tool included ten questions that were divided into six groups: selection bias, performance bias, detection bias, attrition bias, reporting bias, and other sources of bias [40].

A structured list of signaling questions was applied to each item in the tool, leading to a judgment about the risk of bias. A no-signaling question was answered with “yes”, which meant that the likelihood of bias was a low risk, whereas “no” meant high risk. In the case of a study in which the information was not reported adequately to allow a clear judgment, the answer was indicated as “unclear”, indicating that it might have been underreported or the study design was not clear [40]. Two reviewers were going to assess each study independently in order to reduce the subjectivity level, and the difference in scoring was resolved through consensus discussion.

## Results

**Fig 1:**
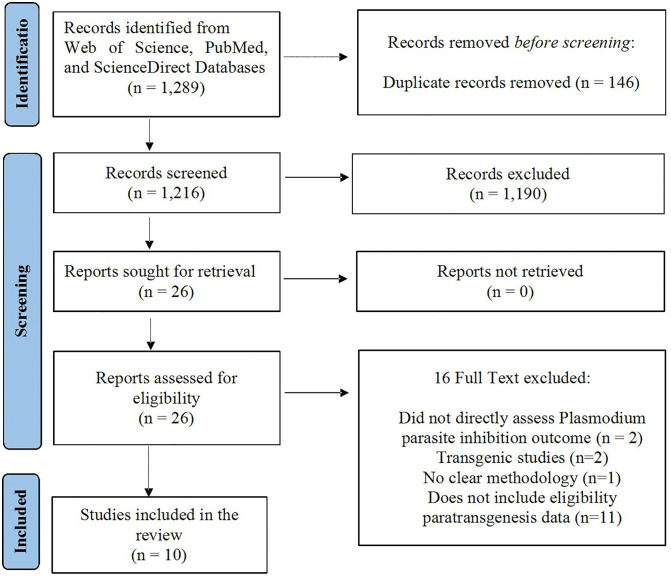
Flowchart of the article search and screening process.

The PRISMA flow diagram illustrates this systematic review’s comprehensive study selection process. First, 1289 records were identified from Web of Science, PubMed, and ScienceDirect databases. Upon the deletion of 146 duplicate records, the researchers filtered 1,216 articles based on their titles and abstracts, and 1,190 articles did not fit the inclusion criteria. All the potentially relevant 26 studies were retrieved and evaluated as eligible. Sixteen were eliminated based on the following criteria: two did not directly determine the inhibition of *Plasmodium* parasites, two were transgenic studies, one did not have a clear methodology, and eleven did not have qualified paratransgenesis data. The final screening gave 10 studies that met the inclusion criteria and were included in the systematic review (Fig 1).

### Paratransgenic strategies targeting *Plasmodium d*evelopment

Several independent studies have investigated the application of paratransgenesis to block malaria transmission, using different effector proteins, microbial hosts, plasmid systems, and mosquito-parasite models. Each study employed distinct experimental designs, ranging from bacterial symbionts such as *Serratia, Asaia, and Enterobacter* to recombinant fungal systems like *Aspergillus oryzae.* Table 1 below summarizes the key features of these studies, including their objectives, geographical settings, mosquito and *Plasmodium* species used, expression systems, effector molecules tested, dosing regimens, and method of parasite quantification.

**Table 1:** Summary of data from the ten selected studies.

Ref	Objective/Aim	Location	Mosquito/ Plamodium Specie	Plasmid Used/	Bacteria/ Fungi Paratransgenized	Effector protein expressed	Bacteria Dose (CFU/ml)	Quantification of the parasite
[41]	Evaluate the transmission blocking potential of wild-type (WT) and engineered strains of E. cloacae	Iran	*Anopheles stephensi/* *Plasmodium berghei*	pBR322	*Enterobacter cloacae*	Scorpine, Defensin, and Has A secretion system	1 x 10^9 CFU/ml	Staining with 0.5% Mercurochrome and counting under the light microscope
[42]	Compare transgenesis and paratransgenesis as genetic strategies for blocking malaria transmission by inhibiting *Plasmodium* development.	USA	*Anopheles stephensi/* *Plasmodium berghei*	QUAS -E plasmid	*Serratia AS1* strain	Scorpine and Midgut peptide (MP2)	1 x 10^7 CFU/ml	Staining with 0.1% Mercurochrome and counting under the light microscope
[43]	To show that the recombinant *A. oryzae* can be used as a paratransgenesis system to inhibit oocyst formation in *An. stephensi*	Turkey	*Anopheles stephensi/* *Plasmodium berghei*	N/A	*Aspergillus oryzae* fungi	MP2 and EPIP	4 × 10^6 conidia/mL	10% Geimsa stain was used, and oocysts were counted using confocal microscopy
[44]	Isolate native Asaia protein secretion signals and construct paratransgenic strains capable of delivering antiplasmodial effector proteins into the midgut of A. stephensi mosquitoes.	USA	*Anopheles stephensi/* *Plasmodium berghei*	pNB97	*Asaia bogorensis SF2.1*	Scorpine and anti-Pbs21- scFv-Shiva 1	1 x 10^8 CFU/mL	Staining with 0.1% Mercurochrome and counting under the light microscope
[45]	To develop a paratransgenic system in Asaia bogorensis SF2.1 that conditionally expresses the antiplasmodial effector protein scorpine only after a mosquito blood meal, using blood meal-inducible promoters	USA	*Anopheles stephensi/* *Plasmodium berghei*	pCG18.glr1	*Asaia bogorensis SF2.1*	Scorpine fused to phoA, having four blood meal inducible promoters: Hem, HF, HlyA, and HlyC	1 x 10^8 CFU/mL	A 100-fold dilution of mercurochrome stain for 2 min, and the oocytes were observed at 100X magnification under a light microscope.
[36]	To show that genetically engineered *Asaia bogorensis* expression effectors proteins can reduce *Plasmodium* oocyst formation	USA	*Anopheles stephensi/* *Plasmodium berghei*	pHyp4s.Myc	*Asaia bogorensis SF2.1*	Scorpine with five fusion proteins: MYC, His, TrxA, GST, and MBP, and Hyp4	1 x 10^8 CFU/mL	Staining with 0.5% Mercurochrome and counting under the light microscope
[46]	To identify native *Asaia bogorensis* signal peptides capable of secreting antiplasmodial effectors	USA	*Anopheles stephensi/* *Plasmodium berghei*	pNB92	*Asaia bogorensis SF2.1*	Scorpine expressing three different signal peptides; Hyp1s, TonBs, and Hyp4s	1 x 10^8 CFU/mL	A 100-fold dilution of mercurochrome stain for 2 min, and the oocytes were observed at 100X magnification under a light microscope.
[47]	To engineer the natural mosquito symbiont *Pantoea agglomerans* to stably express and secrete antimalarial effector proteins in the midgut of *Anopheles* mosquitoes	USA	*Anopheles gambiae/* *Plasmodium falciparum* and *Plasmodium berghei*	pPnptII:gfp	*Pantoea agglomerans*	Scorpine, EPIP4, Pro-EPIP, Shiva1, mPLA2	1 x 10^9 CFU/ml	Manual counting of oocysts under a light microscope
[48]	To engineer the native mosquito symbiont *Serratia AS1* to express and deliver antimalarial effector proteins within the midgut of *Anopheles gambiae*	USA	*Anopheles gambiae/* *Plasmodium falciparum*	N/A	*Serratia AS1*	HasA, Shiva1, (EPIP)₄, (MP2)₂, mPLA2, Scorpine, Multi (EPIP)₄-Shiva1-(SM1)₂	1 x 10^7 CFU/ml	stained and examined under a light microscope, and *P. falciparum* oocysts were counted manually
[34]	To engineer *Escherichia coli* and *Enterobacter agglomerans* to express anti-*Plasmodium* molecules	USA	*Anopheles stephensi/* *Plasmodium berghei*	pTX215	*Enterobacter agglomerans*	SM1–1, SM1–2 and PLA2		Staining with 0.5% Mercurochrome and counting under the fluorescent microscope

A. pBR/DG, pNB97, pCG18.glr1, pTX215, pPnptII:gfp, pBR322, QUAS-E: Plasmid names (engineered DNA vectors used for gene delivery).

B. MP2 (Midgut peptide) and EPIP (Enolase-plasminogen interaction peptide).

C. Epitope tag (MYC), Polyhistidine tag (His), Thioredoxin (TrxA), Glutathione S-transferase (GST), Maltose-binding protein (MBP), and Scorpine expressing one signal peptide (Hyp4).

D. SM1 variant peptides (SM1–1 and SM1–2) and Phospholipase A2-H67N Mutant (PLA2).

E. N/A: Not applicable, which means it was not clearly stated in the study.

Across the reviewed studies, the timing of mosquito dissection ranged from 8 to 14 days after infection, depending on the mosquito and *Plasmodium* species used. The Majority of the researchers who worked with *Plasmodium berghei* waited 10–14 days to enable the parasite to develop visible oocysts in the midgut of the mosquito. Studies conducted by Dehghan and Kianifard [41,49] performed dissections 10–12 days after infection, whereas Grogan and Bongio [36,44] conducted them up to 14 days to ensure full parasite development. Conversely, in the case of working with P*lasmodium falciparum*, the researcher Wang [47] dissected earlier, around 8 days, perhaps to see the early infection stages. These variations reveal the influence of the biology of both the mosquito and the parasite on the design of experiments in both instances [36,41,49,44].

In each of the investigated papers, oral feeding was used as the method of delivering genetically modified symbionts to the mosquito midgut, which was usually in the form of a sugar meal. This pattern is indicative of its usefulness and affordability to administer engineered microbes to adult mosquitoes. The bacteria and fungi were inoculated in a 5% solution of sucrose and incubated overnight, a procedure that simulates the natural feeding behaviour of mosquitoes and gives the opportunity to accurately colonize them. Conidial suspensions were administered even in fungi [49], where the formulation was prepared so that it could be ingested. The existence of sugar meal feeding in a wide range of microbial strains and mosquito *Plasmodium* models highlights the standardization of sugar meal feeding as the standard method in experiments on paratransgenesis, a non-invasive and reproducible system of delivery of symbionts.

The compiled studies reveal that scorpine emerged as the most widely utilized effector protein in paratransgenic approaches for malaria control, demonstrating consistent and potent antiplasmodial activity across multiple microbial systems. The most consistent and potent effects were observed with the scorpine effector, which achieved up to 97.8% inhibition of *P. falciparum* oocysts, followed by 97.7% inhibition of EPIP4 (Enolase-plasminogen interaction peptide) expression protein when delivered by *Pantoea agglomerans*, using pPnptII:gfp plasmid, that expressed green fluorescent protein (GFP) [47]. Similarly, a study conducted by Wang [48] showed that scorpine had a 93.0% oocysts inhibition of *P. falciparum* when expressed in *Serratia AS1*.

Another study showed that *Asaia bogorensis* SF2.1, engineered to express the antiplasmodial protein Scorpine, only after a blood meal (using inducible promoters), significantly reduced *P. berghei* infection in *Anopheles stephensi*. The engineered strains lowered counts to 2 and reduced prevalence to 58.6%–73.7%. Blood meal-induced Scorpine expression proved more effective and sustainable, balancing antiplasmodial activity with bacterial fitness [50].

Grogan [36,46] systematically optimized *Asaia bogorensis* SF2.1 for paratransgenic malaria control by enhancing Scorpine secretion and activity. In 2021, they demonstrated that native signal peptides (Hyp1s/Hyp4s) enabled efficient Scorpine secretion, reducing *Plasmodium berghei* oocysts from 18 (wild-type) to 3, while lowering prevalence to 75.6% and 70.2% respectively. Building on this, their 2022 study tested fusion partners (TrxA, GST, MBP) with Hyp4s, revealing GST-Scorpine and TrxA-Scorpine as top performers, cutting oocysts to 3 (prevalence: 68.6 and 78.7% respectively), comparable to the positive control PhoA-Scorpine (2 oocysts, 67.2%). MBP-Scorpine was moderately effective (5 oocysts). Together, these studies identified Hyp1s/Hyp4s signal peptides and TrxA/GST fusions as optimal tools to maximize Scorpine’s antiplasmodial effects while maintaining bacterial fitness, providing a dual-strategy framework for engineering more effective paratransgenic *Asaia strains*.

In line with these findings, another independent study that performed Asaia-based paratransgenesis in the *Anopheles stephensi* mosquitoes demonstrated that Scorpine decreased *P. berghei* mean oocyst loads by 63.0% [44]. These works highlight the ability of Scorpine to be effective across microbial systems and *Plasmodium* species, establishing it as the most potent and widely used effector in paratransgenesis-based malaria vector control.

Another study by Dehghan [41] also affirmed that Defensin could be utilized as an effective effector protein in paratransgenesis, which demonstrated 92.8% oocyst inhibition with *P. berghei* compared to other expression vectors counterparts Scorpine (92.5%, and Has A (85.8%), when expressed in *Enterobacter cloacae* using the PBR322 plasmid.

A research study conducted by Huang [37] affirmed that paratransgenesis and transgenesis independently provided extensive transmission-blocking capability towards *Plasmodium berghei* in mosquitoes. A rate of 69.3% sporozoite inhibition was reported in paratransgenic mosquitoes colonized with the recombinant *Serratia AS1* strain, which expresses a multi-effector protein (MEP), and a rate of 85.2% was reported in engineered transgenic mosquitoes. A synergistic strategy involving paratransgenesis and transgenesis, and midgut peptide (MP2) and scorpine showed better and more effective results with 89.8% sporozoite inhibition. This dual-strategy used the QUAS-E plasmid system, and it was tested in three independent mosquito infection challenges. This indicates a synergistic process whereby coincidental interference at several life cycle stages of the parasite, including midgut invasion, oocyte development, and sporozoite migration through gut microbiota and transgenic tissue expression proteins.

One of the studies assessed the anti-oocystogenic properties of recombinant *Aspergillus oryzae* on oocyst inhibition of *P. berghei* in female *Anopheles stephensi* mosquitoes. The recombinant strains exhibited high antiplasmodial activity. *A. oryzae-R_E*, which expresses the EPIP effector molecule, inhibited oocyst development by 79.6%. Equally, *A. oryzae-R _ M* expressing MP2 showed 83% oocyst inhibition. The interaction with A. oryzae-RE and A. oryzae-RM was the most pronounced, with the synergy indicating 88% oocyst inhibition [49]. These results indicated that the recombinant strains acting on *Plasmodium* had different effects, and their combination was more effective. This study emphasized the potential of engineered *A. oryzae* as a paratransgenic agent to control malaria, especially in the deployment of several effector molecules. Future research is required to investigate the molecular mechanism and optimize delivery for field applications.

Finally, Riehle [34] used paratransgenic method by constructing *Escherichia coli* and *Enterobacter agglomerans* to express anti-*Plasmodium* effectors (SM1 peptidogens, H67N PLA2) and evaluate their effects on *Plasmodium berghei* inhibition in *Anopheles stephensi*. Although no inhibition was observed with control bacteria (Lpp’OmpA), SM1–2 was the strongest single effector with a 41.4% inhibition as compared to SM1–1 with 28.3% and H67N PLA2 with 23.3%. Additive and not synergistic interactions were observed as a combination of H67N PLA2 + SM1–2, resulting in a 39% inhibition. The research presented provided baseline information on paratransgenesis in which the optimization of effector selection and strategic combinations is important in improving the malaria transmission-blocking efficacies.

### *Plasmodium* mean percentage inhibition outcome

**Fig 2:**
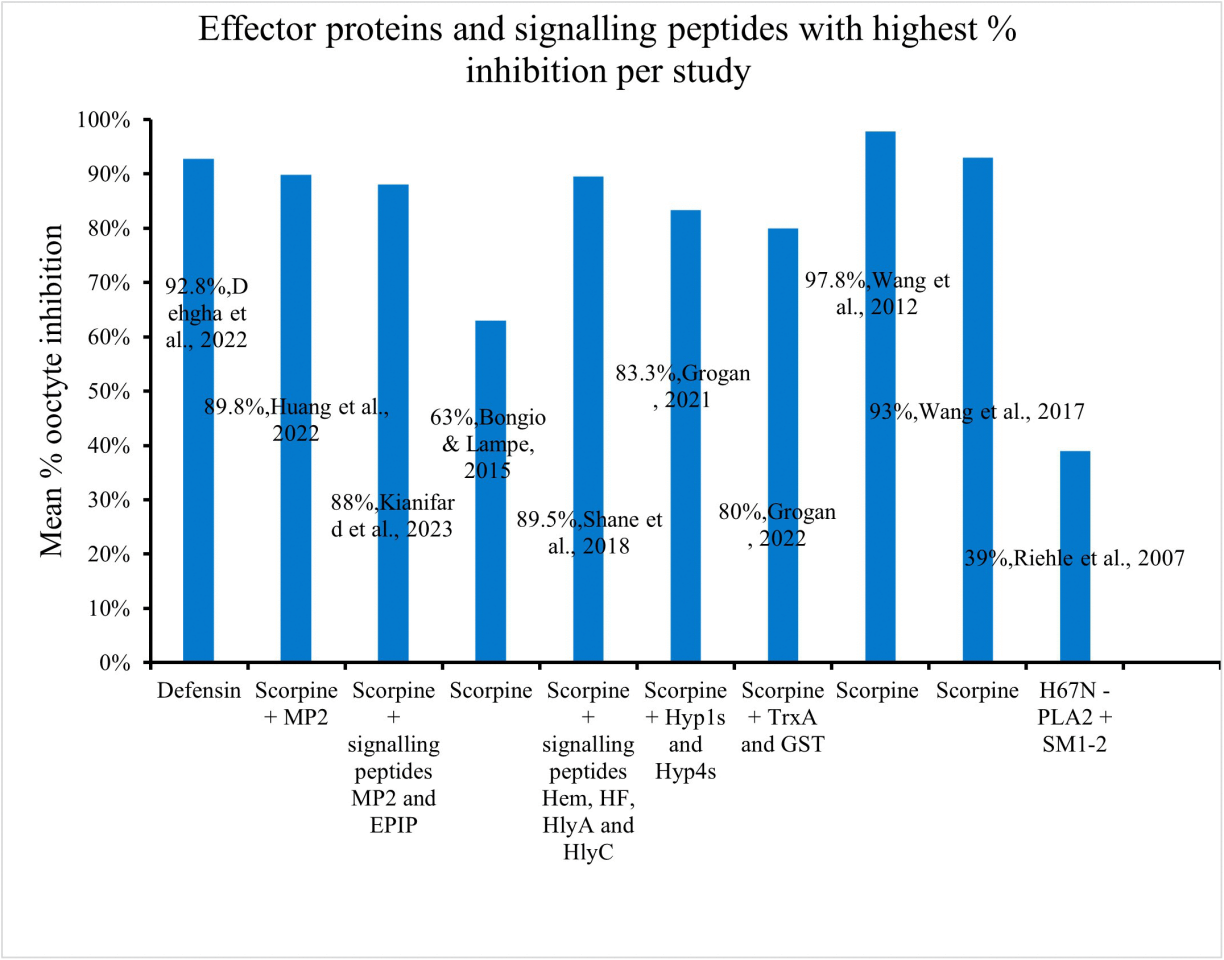
Comparative inhibition of effector proteins and signaling peptides.

A large variety of effector proteins and signalling peptides have been used as paratransgenes to interfere with the development of malaria parasites in mosquitoes. The strengths of these effectors differ with some recording small decreases in *Plasmodium* infection, and some having almost complete transmission blocking properties. The most successful and consistent effector protein was Scorpine, outcompeting alternative candidates, including Defensin, EPIP, MP2, and SM1–2. The comparative inhibition outcomes of these effectors are illustrated in (Fig 2) below.

### Risk of bias analysis

**Fig 3:**
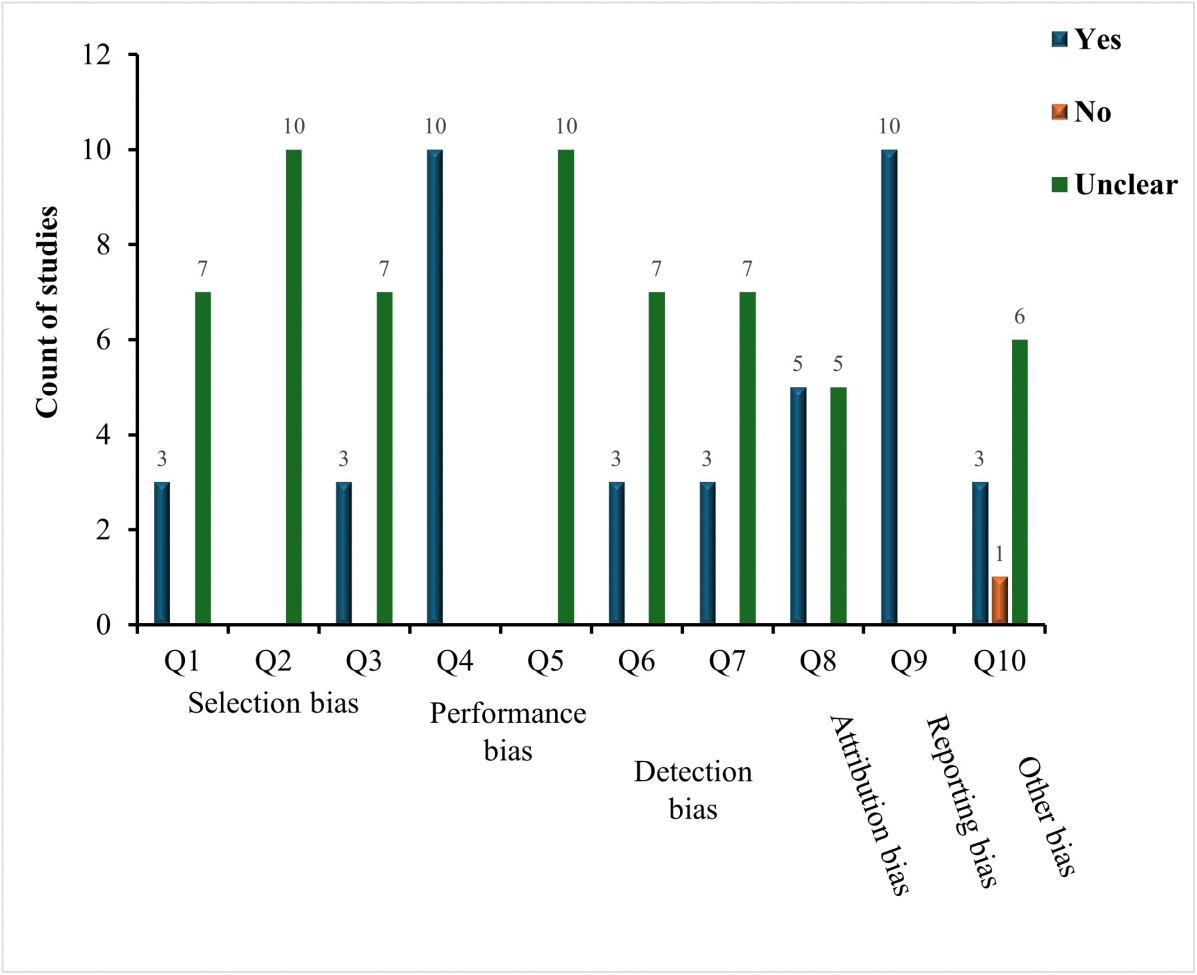
Summary of findings of risk of bias assessment.

The outcomes of the risk of bias evaluation are presented in a multiple bar chart (Fig 3), providing an overview of study quality and informing the confidence in the synthesized findings of this systematic review, particularly regarding reproducibility and potential sources of experimental variation in mosquito paratransgenesis studies. Selection bias was assessed through three questions: Q1 evaluated whether mosquitoes were randomly allocated to experimental groups, with 3 studies rated low risk (Yes) and 7 studies unclear; Q2 examined baseline similarity between groups or control for confounders, which was unclear in all 10 studies; and Q3 assessed allocation concealment, with 3 studies low risk and 7 unclear. Performance bias was assessed by Q4, which determined whether mosquitoes were housed randomly or physically separated to avoid cross-contamination. All 10 studies were low risk, and Q5, which considered whether experimenters were blinded to group assignments, was unclear for all studies. Detection bias included Q6, assessing whether mosquitoes were randomly selected for infection measurement (3 low risk, 7 unclear), and Q7, evaluating blinding of outcome assessors during parasite quantification (3 low risk, 7 unclear). Attrition bias (Q8) explored incomplete data, including deaths of the mosquito or failure of dissection, with 5 studies having low risks and 5 that were unclear. Reporting bias (Q9) assessed the complete and clear reporting of all expected effects, and all 10 studies were low risk. Lastly, other biases (Q10) considered additional sources, including variability in microbial dose, colonization confirmation, and experimental reagent; 3 studies were low risk, 1 was high risk, and 6 were unclear.

## Discussion

The systematic review was a review of ten original studies, all researching the potential of paratransgenesis as a new method for controlling vectors in development or the inhibition of *Plasmodium* in Anopheles mosquitoes. These studies reveal that genetically engineered symbiotic microorganisms, especially bacteria and fungi, can be successfully utilized to deliver antiplasmodial effector proteins to the mosquito midgut such that oocyst or sporozoite development was remarkably suppressed. This transmission-blocking approach offers a promising complement to traditional vector control tools such as insecticide-treated nets and antimalarial drugs, especially in the face of increasing resistance [11,12].

This review discussed paratransgenic approaches to Malaria control and identified the research by Wang [47] as a pioneering work in the engineering of *Pantoea agglomerans*, which is a midgut-dwelling bacterium of *Anopheles gambiae* to express several anti-*Plasmodium* effector molecules. Unlike systems that rely on blood-meal inducible promoters or context-specific regulatory elements, the authors used the PnptII:gtf plasmid system, a powerful bacterial promoter due to its stable and sustained gene expression. Such a design of the promoter ensured that antimalarial effectors would be expressed in all stages of *Plasmodium* development in the mosquito vector, possibly maximizing the exposure and interference with the progression of the parasites.

The transformed *P. agglomerans* secreted a series of effector proteins, such as Scorpine, EPIP4, Pro-EPIP, Shiva, and PLA2, which target different stages or physiological aspects of *Plasmodium* [47,51]. The recombinant bacteria were introduced through oral sugar feeding, which allowed for a non-invasive way to colonize the mosquito midgut, similar to the method employed by Kulkarni [52]. Mosquitoes were dissected 8 days after infection, and quantification of parasites revealed that there were very few oocysts in the infected mosquitoes as compared to controls. This demonstrates the potential of using paratransgenic bacteria as a way of suppressing the development of parasites in vivo and validates the use of effectors delivered under constitutive promoter control.

As with other paratrangenesis studies, several limitations remain. The review is limited by heterogeneity in experimental designs, effector molecules, bacterial strains, and outcome measures, which precluded quantitative meta-analysis. Additionally, most included studies were laboratory-based, which limited the inference on the long-term ecological stability and field applicability of paratrangenesis approaches. Although laboratory-determined reduction in parasite load is a promising method, the translational value of such a method depends on strict ecological, biosafety, and regulatory assessments.

Notably, the selection of the PnptII:gtf promoter stands out. In most earlier reports, the expression of effectors was also time-constrained or spatially confined with promoters sensitive to environmental signals, such as blood meals or infection signals. The benefit of continuous expression is that it has the potential to span both pre- and post-blood meal developmental windows of *Plasmodium* and limit parasite temporal escape. However, constitutive expression can potentially cause metabolic burdens to the bacterial host, potentially negating bacterial fitness or colonization efficacy, although these effects were not directly measured in the study. The success of this system could have been due to three major innovations: (1) the potent, constitutive PnptII promoter that facilitated continuous expression of the effectors during the development of the parasite [51]; (2) the native mosquito gut symbiont *P. agglomerans* that ensured stable colonization without rejection by the host immune system [20,53]; (3) built-in GFP that provided real time monitoring of bacterial persistence and protein localization [54].

The research aligns with current studies, which focus on the idea of multi-effector strategies, whereby combining proteins in various stages (e.g., midgut invasion, oocyst maturation, or even sporozoite development) can lead to increased efficacy and reduced chances of resistance. The localization of effector proteins, the efficiency of secretion, and target specificity are, however, important factors affecting the outcome [55].

In the future, field-based validation and modeling of release dynamics, bacterial persistence, and host-pathogen-microbiota interactions will be crucial in determining how to integrate paratransgenesis into malaria vector containment programs. Further studies are also required to evaluate how this approach interacts with native microbiome compositions of field mosquitoes and whether these engineered bacteria can establish and compete effectively in the natural midgut microbial community.

The experiment identified the important design characteristics of paratransgenic vectors: the strength of promoters and bacterial host compatibility are as important as effector selection. The operation of the pPnptII system indicates that the next generation platforms should include: (a) powerful constitutive or stage-specific promoters, (b) native mosquito symbionts as delivery vehicles, and (c) quality control reporter systems. This study provides the basis for future optimization of multi-effector systems in later *Asaia*-based experiments, showing how the potential of transmission blocking can be optimized by strategic plasmid engineering.

Future research areas can focus on: 1) field trials in malaria-infested areas, 2) the creation of fail-safe controls to avoid unintended uptake of microbes, 3) optimization of cocktails of effectors to make them parasite-resistant, and 4) combination with other ways of controlling the problem, such as insecticides or gene drives. The overall high-level inhibition (usually > 90%) obtained in several independent studies [41,47,48] is solid evidence of the concept that paratransgenesis has the potential to be a useful element of malaria eradication efforts. However, as noted by Riehle [34] in early work, transitioning from laboratory success to field application requires addressing complex ecological and implementation challenges.

## Supporting information

S1 Checklist PRISMA 2020 checklist [39]. (DOCX)
